# LSD increases sleep duration the night after microdosing

**DOI:** 10.1038/s41398-024-02900-4

**Published:** 2024-04-15

**Authors:** Nathan Allen, Aron Jeremiah, Robin Murphy, Rachael Sumner, Anna Forsyth, Nicholas Hoeh, David B. Menkes, William Evans, Suresh Muthukumaraswamy, Frederick Sundram, Partha Roop

**Affiliations:** 1 Faculty of Engineering, University of Auckland, Auckland, 1010 New Zealand; 2 School of Pharmacy, Faculty of Medical and Health Sciences, University of Auckland, 85 Park Road, Grafton, Auckland 1023 New Zealand; 3 Department of Psychological Medicine, Faculty of Medical and Health Sciences, University of Auckland, 85 Park Road, Auckland, 1023 New Zealand; 4 Mana Health, 7 Ruskin St, Parnell, Auckland, 1052 New Zealand

**Keywords:** Human behaviour, Pharmacodynamics

## Abstract

Microdosing psychedelic drugs at a level below the threshold to induce hallucinations is an increasingly common lifestyle practice. However, the effects of microdosing on sleep have not been previously reported. Here, we report results from a Phase 1 randomized controlled trial in which 80 healthy adult male volunteers received a 6-week course of either LSD (10 µg) or placebo with doses self-administered every third day. Participants used a commercially available sleep/activity tracker for the duration of the trial. Data from 3231 nights of sleep showed that on the night after microdosing, participants in the LSD group slept an extra 24.3 min per night (95% Confidence Interval 10.3–38.3 min) compared to placebo—with no reductions of sleep observed on the dosing day itself. There were no changes in the proportion of time spent in various sleep stages or in participant physical activity. These results show a clear modification of the physiological sleep requirements in healthy male volunteers who microdose LSD. The clear, clinically significant changes in objective measurements of sleep observed are difficult to explain as a placebo effect. Trial registration: Australian New Zealand Clinical Trials Registry: A randomized, double-blind, placebo-controlled trial of repeated microdoses of lysergic acid diethylamide (LSD) in healthy volunteers; https://www.anzctr.org.au/Trial/Registration/TrialReview.aspx?id=381476; ACTRN12621000436875.

## Introduction

The relatively recent emergence of the practice in which users self-administer psychedelic drugs, mainly lysergic acid diethylamide (LSD) or psilocybin, in repeated doses at levels below the threshold for overtly causing hallucinations is termed microdosing [1]. Microdosers claim that this practice can improve mood and well-being, reduce the symptoms of both anxiety and depression, as well as potentially enhance creativity and productivity [2–6]. While the veracity of many of these claims has yet to be adequately tested in randomized controlled trials in clinical populations [7], it is becoming increasingly clear that microdosing can cause distinct changes in neurophysiological function with reported changes in both resting and task-based EEG [8–10] and fMRI-based connectivity [11] but there are no reports of changes in objectively measured behaviors. The potential effects of microdosing on sleep behavior have not yet been thoroughly investigated. In fact, the effects of even macrodoses of psychedelic drugs on sleep function have received only sporadic investigation in the literature over the years. In a study of the N,N-dimethyltryptamine (DMT) containing brew ayahuasca [12], healthy male participants (*n* = 22) were administered placebo, ayahuasca (1 mg DMT/kg), or d-amphetamine in the daytime in a crossover design with polysomnography recorded the night of administration. Ayahuasca did not subjectively impair sleep quality but decreased the amount and relative proportion of time spent in REM sleep, increased REM onset latency, and enhanced power of slow-wave NREM sleep. Similarly, in another crossover design study [13], participants (*n* = 20) were administered either psilocybin (0.26 mg/kg) or a placebo in the morning, with polysomnography recorded in the evening. Results showed that psilocybin increased REM onset latency with a trend to decreased REM duration, while sleep latency, total sleep time, and the number of sleep cycles were not affected. These human studies concur with animal studies of the serotonergic psychedelics LSD, 2,5-Dimethoxy-4-iodoamphetamine (DOI), and mescaline, which have been found to generally increase wakefulness and decrease both REM and NREM durations [14–16].

With respect to microdosing, there is only a single report of objective sleep measurements available. Published in 1966, Muzio et al. [17] administered participants low doses of LSD (range 4–40 µg) just prior to or one hour after participants went to sleep by briefly awakening them to administer the drug. Generally, LSD was found to significantly increase REM duration, with REM bursts interrupting slow-wave sleep as well as increased body movements and arousal periods occurring during some REM sleep periods. However, while the doses delivered in this study do overlap with the modern microdosing range, the timing of drug administration relative to sleep is quite inconsistent with modern microdosing practices, which generally involve daytime microdosing.

Several microdosing studies have provided some subjective reporting around sleep quality. These studies tend to show bidirectional effects, with both improvements and difficulties in sleeping reported. Interviews with a small sample (*n* = 24) of community microdosers found that overdosing and insomnia are common challenges of microdosing [18]. Similarly, in an online survey of 525 participants who were currently microdosing, 45% reported having had trouble sleeping ever, with 3.2% saying it occurred often [5]. On the other hand, a large cross-sectional study of community microdosers (*n* = 3933) endorsed improving sleep as a motivation for microdosing [6]. Consistent with this, in an online cross-sectional survey (*n* = 278) of microdosers, 28% of respondents found that microdosing improved their sleep quality [2]. In a crossover study of participants using psilocybin-containing truffles, modifications to sleep patterns were noted by a small subset of participants [19].

Overall, given the paucity of information regarding the effects of microdosing on objective sleep measures, in our recent Phase 1 microdosing trial [20] using home administration of LSD microdoses (10 µg base) in ecologically valid settings, all participants were given commercially available watches to wear to enable monitoring of sleep and activity patterns. The sensors in consumer-grade devices have been shown to be useful in assessing sleep stages, sleep-wake states, and REM sleep in naturalistic settings [21, 22]. Participants microdosed at home every third day for six weeks with a 1-week baseline run-in period. Microdosing every third day is a commonly used pattern as originally described by Fadiman [1]. The rationale for this approach is to avoid tolerance effects as LSD was known since the 1950s to produce tolerance and reversal effects in just a few days [23, 24], although tolerance in the context of microdosing has not been explicitly tested. Given that we planned to have 80 participants engaged in the protocol for approximately 49 nights, with fourteen doses per participant we had potentially 3920 sleep nights in the trial that could be analysed. Surprisingly, the results demonstrated that LSD microdosing caused robust and significant changes in REM sleep duration and total sleep duration the night after a microdose—with no effects observed on the night of the microdose.

## Methods

### Study design

The design of the current (acronym: MDLSD) study was a double-blind parallel-groups trial. Healthy male volunteers were randomized into LSD (*n* = 40) and placebo (*n* = 40) groups and self-administered 14 doses of either 10 µg LSD base (in water for injection) or inactive placebo (water only) by 1 mL oral syringe for sublingual administration every three days for 6 weeks. The study consisted of four visits for each participant: a screening visit at which they were assessed for eligibility, a baseline visit, followed by a first dosing visit (day 1) session seven days after the baseline visit, and a final follow-up visit scheduled two days after the final microdose (day 42). Full protocol methods were prospectively published prior to any study visits occurring [25], and a full description of the conduct of the trial has been previously published [20]. Only details relevant to the current results are recapitulated here. Ethics approval was awarded by a New Zealand Health and Disability Ethics Committee (19/STH/91), and the HRC Standing Committee on Therapeutic Trials (Online reference: 19/SCOTT/108), and informed consent was obtained from all participants. The trial was prospectively registered at the Australian New Zealand Clinical Trials Register (ANZCTR) reference ACTRN12621000436875.

### Participants

In total, 80 healthy male participants were enrolled in the trial, with 40 participants randomized into the LSD group and 40 into the placebo group (see [20] for CONSORT diagram). Key inclusion criteria included being male between the age of 25–60. Key exclusion criteria included: resting blood pressure over 160/90 mmHg, body weight <50 kg or >120 kg, significant renal or hepatic impairment, unstable medical or neurological conditions, lifetime history of depression/schizophrenia and psychotic disorders or current diagnosis of anxiety or eating disorders, suicidality, first degree relatives with a psychotic disorder, substance use disorder, use of psychotropic medication, use of a serotonergic psychedelic in the last year and any lifetime history of psychedelic microdosing. A urine drug test was performed at screening, and a breathalyzer test was performed at the baseline and first dosing visits. Demographic characteristics of the population sample are shown in Supplementary Table 1.

### Procedures

Nearing the end of the baseline measurement session, participants were given Fitbit Charge 3/4 devices and instructed to try to wear them for the remainder of the trial (apart from when they needed to charge the device or if it caused discomfort). The Fitbit app was installed on participant’s mobile devices to allow data synchronization via Bluetooth. All notifications from the Fitbit app were turned off except for the low-battery reminder. Typically the device required charging once per week. On dosing days participants were asked to dose before 11am to avoid any potential disruption to their sleep. Starting at 7 am, participants received their first SMS reminder to take their microdose. They received hourly reminders until dose completion. For verifying dose compliance, participants were asked to video themselves taking the dose which they then uploaded to the study database. These videos were checked by the study team and then deleted [20, 25]. The video upload timestamps were used to establish the time of day each dose was taken.

### Data analysis

The dataset for this study was initially available in JSON format and converted to text format using Python for easier manipulation and processing. Fitbit separates sleep into 4 states: “REM”, “Deep”, “Light”, and “Awake,” where “Light” corresponds to polysomnography stages N1 + N2, “Deep” is stage N3, “REM” is REM sleep and “Awake” is Wake After Sleep Onset (WASO) [26]. We computed variables “Asleep” as equal to (Deep + Light + REM) and total as equal to (Deep + Light + REM + Awake)—the latter of which is similar to Total Sleep Time in polysomnography. The Fitbit sleep data was split across two files, presenting the potential for overlapping dates. 20 entries were identified with duplicate information, which were subsequently removed for accuracy. The sleep data supplied by Fitbit offered two methods to discern total sleep times: “Sleep Summary” and “Sleep Granular”. The Sleep Summary, gives the total time in minutes of each sleep state for each sleep. In contrast, the Sleep Granular data provides the duration and type of sleep transitions. An examination was performed of both methods to identify any discrepancies. The difference between the two sets was evaluated using the formula ${\rm{Error}}=\frac{\left|\mu -A\right|}{\mu }$, where $\mu =\frac{A+B}{2}$ and $A$ and $B$ represent data from the summary and granular datasets, respectively. The analysis revealed a mean error of 2.11 and a maximum error of 11.37 min for total REM sleep. Given the relatively small error magnitude, we opted to utilize the summary data for subsequent analysis, but analysis of the granular data shows the same pattern of results presented here.

**Fig. 1:**
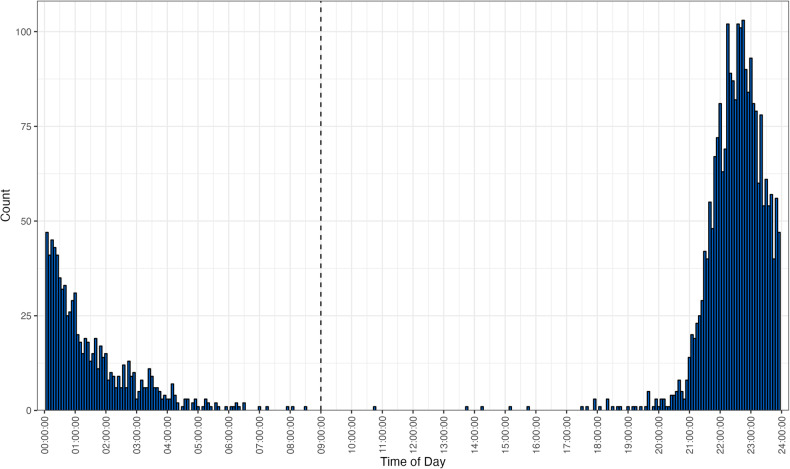
Distribution of participant sleep start times across the trial, grouped into 5-min chunks. The cut-off time for a night’s sleep is indicated by the dashed line at 9:00 am.

It is noteworthy that Fitbit assigns sleep data based on the sleep start date. A cursory assumption might suggest that the start sleep date corresponds accurately with the dosing days, but this was not always the case. To ensure accurate sleep date assignment, all sleep start times were plotted, as shown in Fig. 1, to determine an appropriate “cut-off” time. The graph suggests that an appropriate cut-off time would be 9 am, which roughly matches up with the expected microdosing time, where any sleep events before that would correspond to the previous night.

Finally, the sleep data provided by Fitbit consists of a variety of sleep times. The primary interest was in analyzing the effects of LSD on sleep and focusing on how it affects a person’s main sleep, not any napping. As such, we employed Fitbit’s “isMainSleep” flag to filter out nap times. This flag is set to true if the sleep is the main sleep of the day and false if it is a nap. Notably, Fitbit may assign two main sleep periods if the main sleep is interrupted by a significant period of wakefulness, such as a trip to the toilet—these occurrences were merged.

### Statistical methods

Statistical analyses were conducted using linear mixed-effects modeling using the lmerTest package in R [27] with Group (LSD, placebo) and Day (Dose, Dose + 1, Dose + 2) being treated as fixed effects with dummy coding (with the dosing day as the baseline condition), and participants as a random effect. The primary estimate of interest is the Group × Day interaction effect. All data from the trial was analyzed using an intention-to-treat scheme with no imputation for missing data due to participant dropouts or missing sleep/activity data. There was a baseline period of seven nights of sleep, the average of which was used as a covariate in the analyses. Although the protocol was published prospectively, we had no pre-specified analyses or hypotheses relating to sleep, and as such, the analyses here should be considered exploratory. To correct for multiple comparisons, Bonferroni correction was applied to the alpha threshold for each analysis conducted depending on the number of comparisons conducted within an analysis. Due to the simplicity of this process, the code has not been made available online but is available on request.

## Results

Of the 80 participants who commenced the trial, 5 did not complete the study protocol. Four were discontinued from the trial due to adverse effects (mild anxiety), and one for reasons unrelated to the trial. A further three participants received an extra dose due to scheduling issues with follow-up and were in trial for an additional 2–4 nights. In Supplementary Fig. 1, the total number of nights of sleep data are plotted for each participant. Overall, there were 3231 nights of data available for analysis, including 503 nights of baseline data, 935 nights of dosing day data, 927 nights the day after dosing, and 866 two nights after microdosing. The mean number of nights of data available per participant was 40.39 (sd = 9.96).

**Fig. 2:**
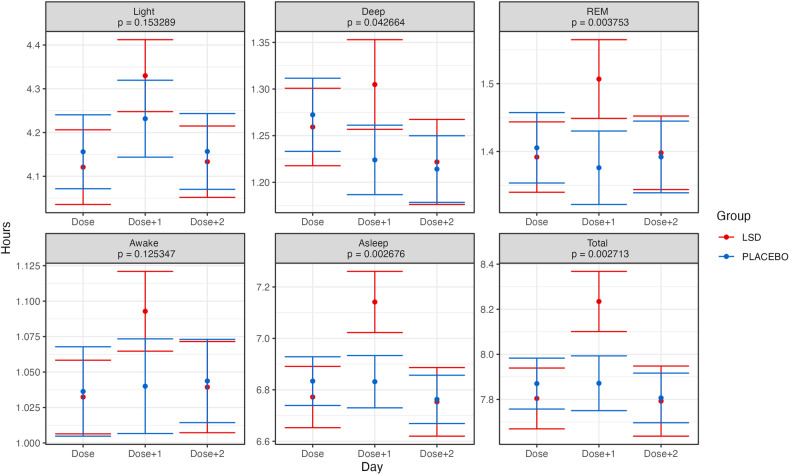
Grand average time spent in each of the sleep stages for each day (dose, dose + 1 dose + 2) and group in the trial. Error bars represent the standard error of the mean calculated across participants. Presented *p* values are for the Group × Day interaction effect. The Bonferroni-corrected alpha threshold for these analyses was 0.00833 (0.05/6). Note: Asleep = Deep + Light + REM. Total = Deep + Light + REM + Awake. See text for details.

**Fig. 3:**
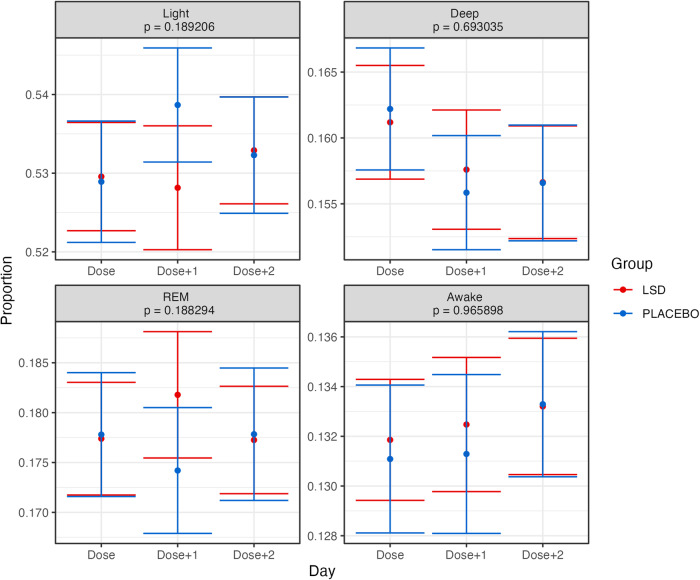
Grand average distribution of each sleep stage (as a proportion of total sleep) for each day (dose, dose + 1 dose + 2) and group in the trial. Error bars represent the standard error of the mean calculated across participants. Presented *p* values are for the Group × Day interaction effect. The Bonferroni-corrected alpha threshold for these analyses was 0.0125 (0.05/4). See text for details.

Figure 2 presents the results of the linear mixed model analysis, with baseline adjustment, of time spent in each stage (Deep, Light, REM, Awake) as well as Asleep and Total. All mixed model residuals were tested for normality. The overall pattern of effects showed a relatively increased time for all metrics on the Dose + 1 night, but only time spent in REM sleep (*p* = 0.0037), Asleep (*p* = 0.0026), and Total (*p* = 0.0027) reached significance with the Bonferroni corrected significance threshold of 0.00833 (0.05/6). These differences corresponded to an extra 8.13 min of REM sleep (95% CI 3.34–12.9), 21.1 min Asleep (95% CI 8.9–33.2), and 24.3 min of Total sleep (95% CI 10.3–38.3) on the Dose + 1 night for the LSD group, compared to the Placebo group. The only metric that met the uncorrected significance threshold of *p* = 0.05 but did not meet the Bonferroni corrected threshold was the amount of time spent in Deep sleep (*p* = 0.043). Similar results were observed when the average of the baseline run-in period was not included as a covariate in the analysis (see Supplementary Fig. 2). Given this pattern of results, we then examined the proportion of time spent in each sleep stage—the results of which are displayed in Fig. 3. These analyses showed that there were no changes which approached significance even with an uncorrected significance threshold of *p* = 0.05.

**Fig. 4:**
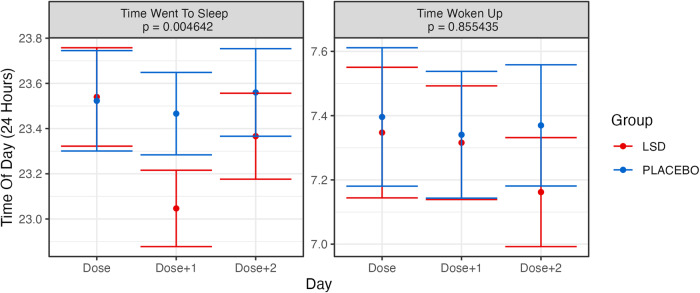
Grand averages for when participants went to sleep and woke up for each day (dose, dose + 1 dose + 2) and group in the trial. Error bars represent the standard error of the mean calculated across participants. Presented *p* values are for the Group × Day interaction effect. The Bonferroni-corrected alpha threshold for these analyses was 0.025 (0.05/2). See text for details.

The observed pattern of sleep alterations was further explored by computing the time that participants went to sleep each night and the time that they woke up the next morning. These results (Fig. 4) demonstrated that participants in the LSD group went to bed significantly (*p* = 0.005) earlier on the Dose + 1 night. This corresponded to going to bed an extra 25.17 min earlier on the Dose + 1 night. No significant change was observed in the time at which participants woke up. It was also tested whether the observed modification in sleep time on the Dose + 1 night changed across the time by adding dose number (1–14) to the mixed model. No effects across time were observed (see Supplementary Fig. 3).

**Fig. 5:**
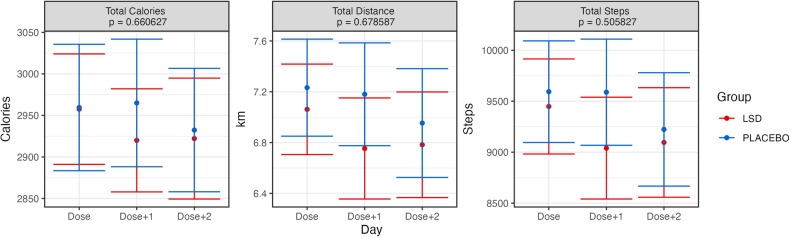
Grand average values for basic activity for each day (dose, dose + 1 dose + 2) and group in the trial. Error bars represent the standard error of the mean calculated across participants. Presented *p* values are for the Group × Day interaction effect. The Bonferroni-corrected alpha threshold for these analyses was 0.013 (0.05/3). See text for details.

**Fig. 6:**
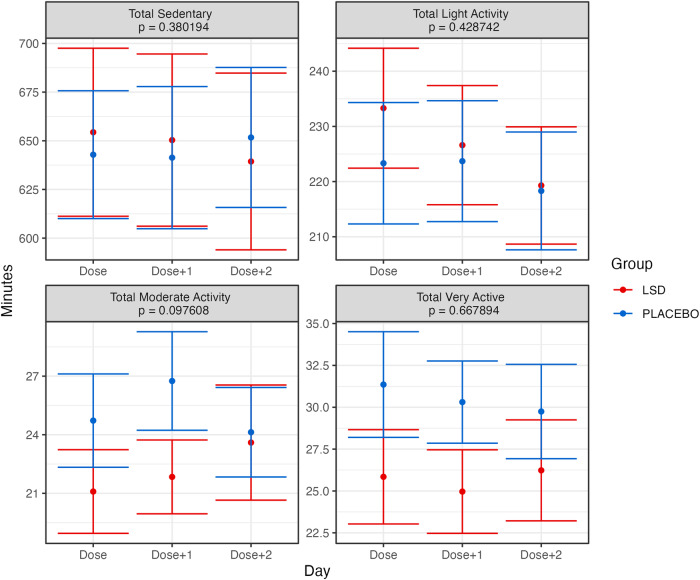
Grand averages of minutes spent in various states of activeness, as categorized by Fitbit, for each day (dose, dose + 1 dose + 2) and group in the trial. Error bars represent the standard error of the mean calculated across participants. Presented *p* values are for the Group × Day interaction effect. The Bonferroni-corrected alpha threshold for these analyses was 0.0125 (0.05/4). See text for details.

One possibility that might explain the extra Dose + 1 sleep requirement was if the physical activity patterns of participants were modified by the drug. Similar to Supplementary Fig. 1, Supplementary Fig. 4 plots the total days of activity data available for analysis for each participant. There was slightly more activity data available for analysis than sleep data. Overall, there were 3842 days of activity data available for analysis, including 556 days of baseline data, 1102 days of dosing day data, 1101 days the day after dosing and 1083 two days after microdosing. The mean days of data available per participant were 45.67 (sd = 6.62). In Fig. 5, various activity pattern metrics (calories, distance, and steps) are plotted. These results show no noticeable or significant changes in interaction effects. Furthermore, we also analyzed the activity state provided by the wearable device in Fig. 6 (sedentary, light activity, moderate activity, very active). As with the previous analysis, there were no significant interaction effects. Visually, participants in the LSD group appear to have overall lower moderately and very active minutes. However, this was also visible in the group’s baseline averages, and there was no main effect.

Although we did not include any subjective measurements of sleep quality, participants were asked to report their tiredness each night and were offered the chance to volunteer pertinent information in their daily report form and in a qualitative interview. Previously we reported a marginally significant effect [20] where participants reported being more tired on Dose + 1 days. Participants could also report sleep disturbances as an adverse event. 10 sleep-related adverse events were reported in the LSD group and 4 in the placebo group—which was not statistically significantly significant in terms of odds ratios [20]. Finally, at the end of the trial, participants took part in a semi-structured interview, the full content of which we plan to publish elsewhere. Relevant to the current report, a number of participants in the LSD group commented that microdosing could both increase and decrease their energy levels (see Supplementary Materials for selected quotes). However, participants in the LSD group did not explicitly comment on needing extra sleep or going to bed earlier.

## Discussion

In this study, it was found that participants in the LSD group had significantly increased sleep time compared to participants in the placebo group when they had taken a microdose the previous day, but no differences were found the night of the dose. These differences corresponded to an extra 8 min of REM sleep, 21 min of asleep time, and 24 min of total sleep time the night after microdosing, with no differences in sleep on the microdosing day itself, with participants going to be earlier the night after microdosing. There were no differences in the ratio of time spent in each sleep stage, nor were there detectable differences in the physical activity of participants between the groups or evidence of tolerance/sensitization.

The extra 24 min of sleep obtained by participants on the Dose + 1 night is not only a statistically significant difference but a clinically meaningful difference between the two groups, with 20 min of sleep speculated to be a clinically meaningful difference in terms of sleep duration [28]. Practically speaking, this result has implications for both the design of therapeutic microdosing protocols with LSD and their potential therapeutic mechanism of action. Pragmatically, the unexpected finding of the extra sleep required after microdosing suggests that it is important for microdosing protocols to have at least one day “off” between doses to ensure that patients are well rested/recovered before the next microdose is taken. While participants did report being marginally more tired on the day after dosing, in retrospect, they did not explicitly mention requiring more sleep or going to bed earlier, suggesting that these processes might have been occurring covertly. In line with reports from lifestyle users [5, 18], some participants in this trial did retrospectively report some trouble sleeping on dose days. However, inspection of adverse event data showed that difficulty sleeping was not reported at significantly higher rates than placebo, and our objective data is consistent with this. This is likely because our home-administration dosing protocol required participants to have dosed by 11am to avoid any disruptions to sleep—a strategy that appears to have been largely successful in this study. One potential explanation for the delay of the extra sleep requirement to the night after microdosing might be due to the pharmacology of LSD. LSD has a relatively long half-life (~4 h) as it becomes trapped in the 5-HT_2A_ receptor pore [29, 30], and the subsequent effects on protein signaling cascades, receptor desensitization, and down-regulation may extend well beyond this [31, 32]. It is unclear whether the same day-after effects would be seen with the other commonly microdosed psychedelic drug psilocybin, which has notably shorter duration effects than LSD [33].

Anecdotally, microdosing users [2–6] have consistently reported improvements in depressive symptomology. Modifications to sleep may be a factor that contributes to that effect. Difficulties with sleeping are commonly reported in mood disorders such as major depressive disorder [34, 35] and premenstrual dysphoric disorder [36]. Polysomnography studies have shown that patients with depression show decreases in slow-wave sleep, as well as shortened REM onset latency and total REM sleep time [37]. It is generally thought that sleep difficulties and depression share a bidirectional causal relationship—although randomized controlled trials looking at sleep interventions to improve depression have only yielded mixed results [34]. Synaptic plasticity theories of depression propose that depression is characterized by modifications of the circuitry underlying cortical adaptability. Sleep is a well-known modulator of synaptic plasticity [38], and it is thought that a critical function of sleep, particularly REM sleep, is to support the formation and consolidation of new memories [39]. In a large epidemiological study, patients with depression reported, on average, 40 min less sleep than non-depressed patients [40]. Speculatively, a candidate therapeutic mechanism by which microdosing LSD might improve mood is by restoring sleep and promoting accompanying synaptic plasticity. In contrast to the current findings with microdosed LSD, most antidepressants actually suppress REM sleep, and first-line treatments for depression, such as selective serotonin reuptake inhibitors (SSRIS) can actually decrease sleep continuity, leading to increased levels of insomnia [37]. As such, the potential use of microdosed LSD as an antidepressant may have very different effects on sleep and therapeutic effects in patients with depression than standard antidepressants.

The current study adds to a growing body of knowledge about microdosing. Several studies using uncontrolled microdosing regimens have claimed that most of the anecdotal, subjective effects of microdosing are probably related to participant expectancy and are explainable as placebo effects [8, 41]. Significant effects of microdosing on vital signs have been relatively inconsistent in controlled trials [42–46], but central nervous system penetration of microdoses has been demonstrated multiple times using EEG [8–10] and fMRI [11]. To our knowledge, this might be the first report of the effect of microdosing on an objectively measurable behavioral outcome, and its completely unexpected nature is difficult to explain as a placebo/expectation effect, As described in the introduction, there are no literature reports to suggest a change in sleep time the night after microdosing and similarly, post-study interviews with participants did not reveal they had any expectations around changes in sleep pattern the night after microdosing. In fact, likely, the only expectation participants may have had around sleep was that it might be disrupted on the night of a microdose if they took the dose too late in the day, as this was communicated to participants by the investigators. Further, the null response in the placebo group data shows no indication of a placebo/expectation response.

### Strengths and limitations

The use of a commercially available wearable device to track sleep and activity is both a strength and weakness of the current study. The major strength of using the fitness trackers in this context is the ability to collect a large amount of naturalistic sleep data (3231 nights worth) while being relatively unintrusive for participants compared to spending nights in a sleep laboratory. For example, and by contrast, the psilocybin sleep study of Dudysova et al. [13] included 40 nights of sleep data recorded the night after a large dose of psilocybin was administered. At the same time, the wearable devices used are limited in that they do not provide reliable access to the important clinical metrics of sleep/REM onset latency. The other limitation of commercial wearable devices is the “black box” nature of the transfer functions, from their physiological sensors to the sleep metrics that they provide end-users for analysis. That said, a number of studies have directly compared Fitbit devices to polysomnography, with a meta-analysis of eight studies [47] concluding that they are accurate and reliable in terms of detecting sleep durations and staging, although still inferior to polysomnography. Finally, the current study did not include any subjective scales of sleep quality which would be useful to include in future microdosing studies.

Other limitations of the current study, as we have previously noted, include the use of only healthy male participants with relatively homogeneous ethnic representation [20]. The rationale for using only male participants was to avoid menstrual cycle confounds known to exist in some of the trial primary outcome measures. However, since females are known to sleep longer, have shorter sleep onset latency, and have higher sleep quality and efficiency, generalisability to the female population requires confirmation [48]. Further, the current healthy volunteer sample excluded persons with mental health issues, yet many people who microdose in the community do so to self-treat mental health conditions. For example, in one survey, 39% of the 1102 respondents [49] did so to self-treat a mental health condition. Our results may not generalize to that population of microdosers nor necessarily to those who microdose with psilocybin, which is the other commonly microdosed drug [49]. In general, replication (and extension) of this unexpected result in the literature within the current study population and to other sub-populations will be important future work to ensure the robustness of the effects observed.

## Conclusion

Given the significant modification in total sleep observed here with LSD microdosing and the potential clinical implications, this result provides a strong justification to incorporate wearable devices for sleep monitoring in our Phase 2 trials of LSD microdosing in patients with major depressive disorder which are currently underway [https://www.anzctr.org.au/Trial/Registration/TrialReview.aspx?id=385758]. More generally, the observation that participants who microdose may require extra sleep the following night suggest that taking “off” days between microdosing days is important to allow the brain and body to recover between microdoses.

### Supplementary information

Supplementary Materials

## Data Availability

The raw data has not been made available.
