# Supplementary material for: LSD increases sleep duration the night after microdosing
Source: Transl Psychiatry. 2024 Apr 15;14:191. doi: 10.1038/s41398-024-02900-4 (PMC11018829; doi:10.1038/s41398-024-02900-4)
Supplement: Supplementary file 1 — Supplementary Materials [file 41398_2024_2900_MOESM1_ESM.docx]

**Supplementary Materials**

Supplementary Table 1: Demographics of all randomised participants for both treatment groups in the MDLSD trial

| Observation |  | Placebo | LSD |
| --- | --- | --- | --- |
| Age, *M* (*sd, range*) | | 36.3 (7.2, 25-56) | 37.4 (9.2, 25-56) |
| Weight, kg, *M* (*sd, range*) | | 83.3 (13.7, 55-112) | 85.1 (14.9, 64-129) |
| BMI, kg/m^2^, *M* (*sd, range*) | | 25.9 (1.9, 19-34) | 26.4 (1.9, 20-39) |
| Lifetime serotonergic psychedelic use, Mdn (IQR) | | 2 (0-7) | 3 (0-8.5) |
| Psychedelic naïve, *n* (%) | | 12 (30) | 12 (30) |
| Ethnicity | Asian, *n* (%) | 4 (10.0) | 6 (15) |
|  | Latin American/Caribbean, *n* (%) | 3 (7.5) | - |
|  | Māori, *n* (%) | 2 (5) | 1 (2.5) |
|  | New Zealand European, *n* (%) | 29 (72.5) | 24 (60) |
|  | Other European, *n* (%) | 5 (12.5) | 11 (27.5) |
|  | Pasifika, *n* (%) | - | 4 (10) |
|  | Other, *n* (%) | 3 (7.5) | 1 (2.5) |

*Note:* All participants in this study are male. Ethnicity percentages will add up to more than 100 due to multiple ethnicities reported by single participants. More detailed demographic information is provided in Murphy et al.^20^.

*Supplementary Table 2: Dose guess count and conditional probability for all doses administered in the trial*

|  | Treatment | |
| --- | --- | --- |
| Guess | LSD, count  (conditional probability) | Placebo, count  (conditional probability) |
| LSD | 328 (0.61) | 121 (0.22) |
| Placebo | 104 (0.20) | 202 (0.36) |
| Don’t know | 109 (0.19) | 238 (0.42) |


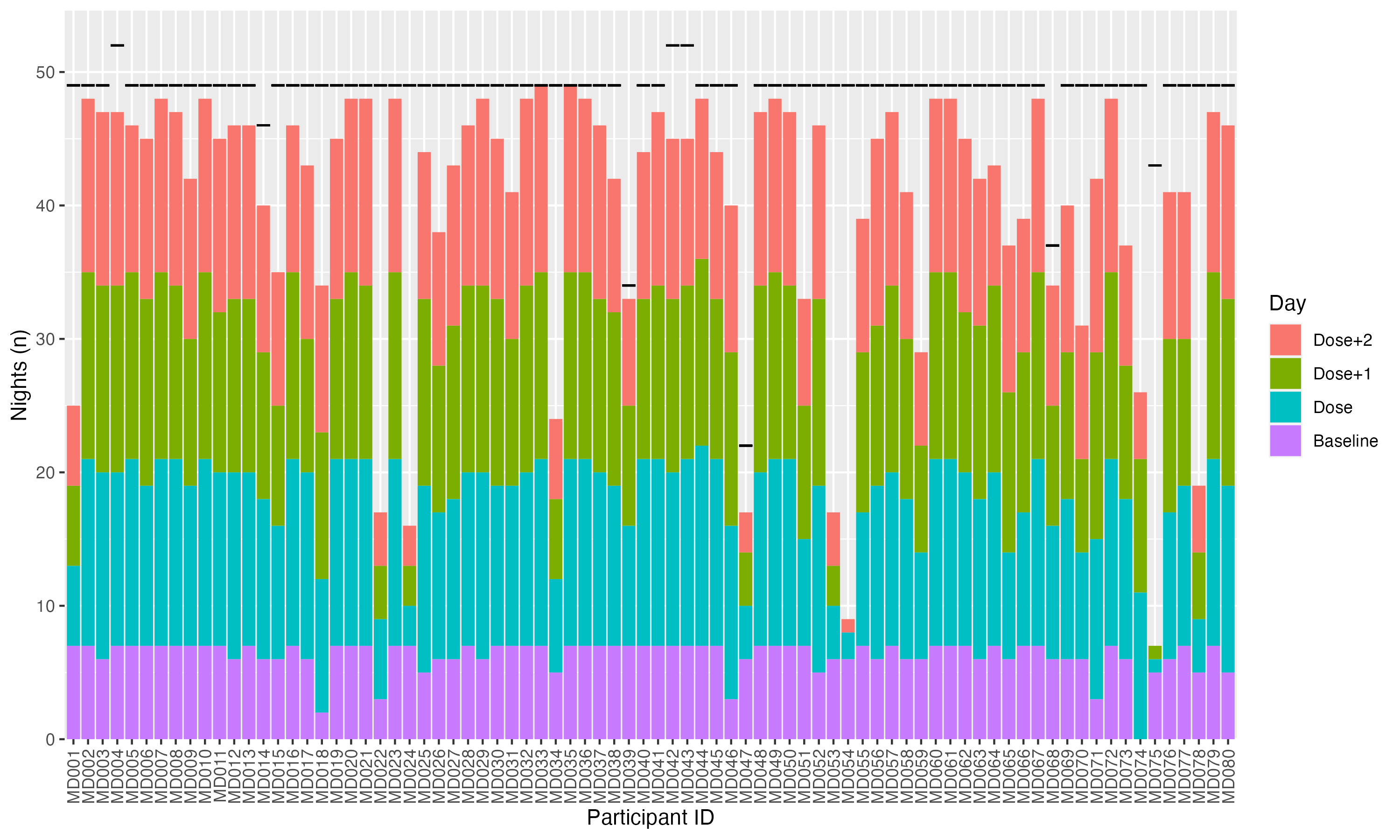


*Supplementary Figure 1: The number of days with quantifiable sleep data for each participant (MD001-MD080) characterised by baseline, dose day , dose+1 day, dose+2 day. See text for details.*


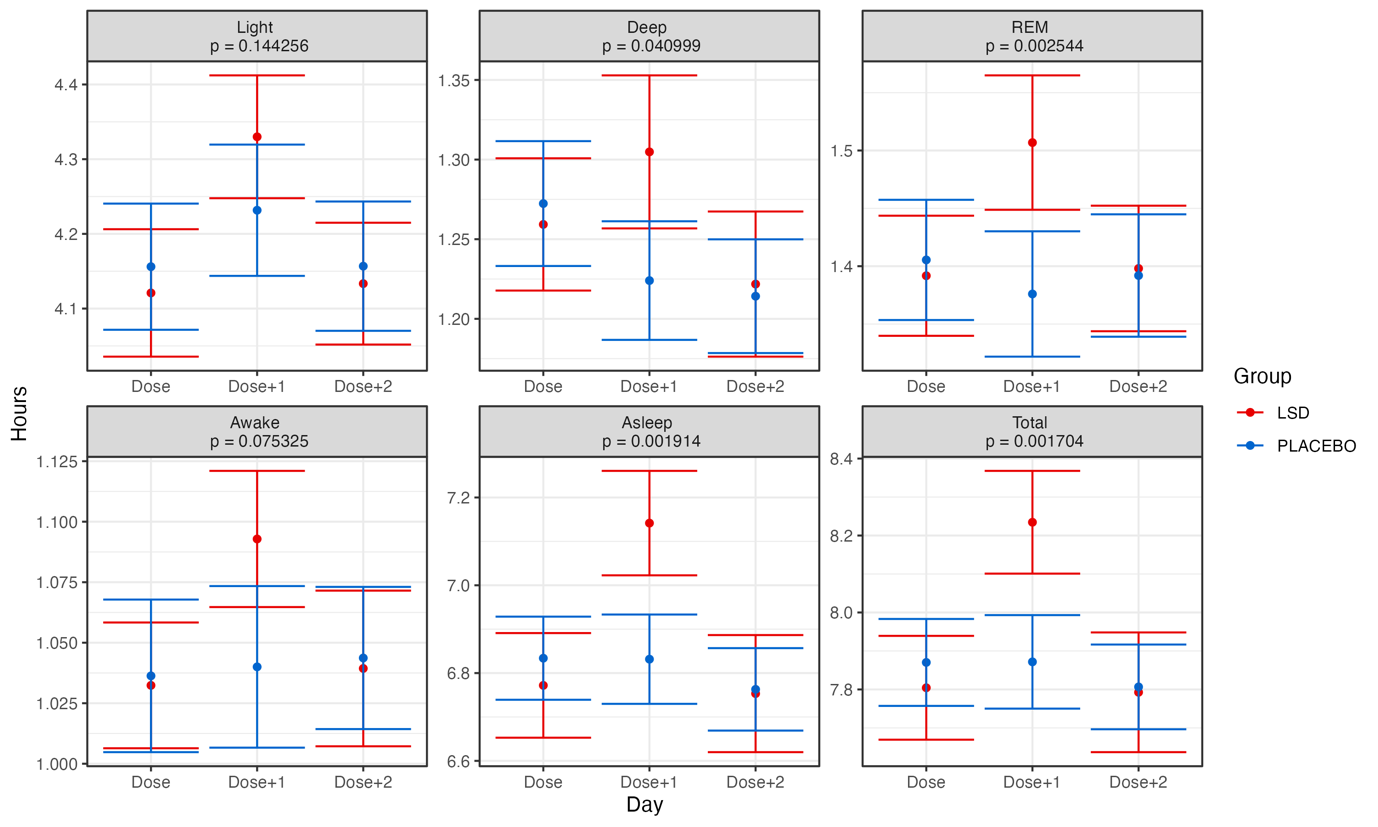


*Supplementary Figure 2: Grand average time spent in each of the sleep stages for each day (dose, dose + 1 dose + 2) and group in the trial. Error bars represent the standard error of the mean calculated across participants. Provided p-values are without baseline adjustment for reference.*

**
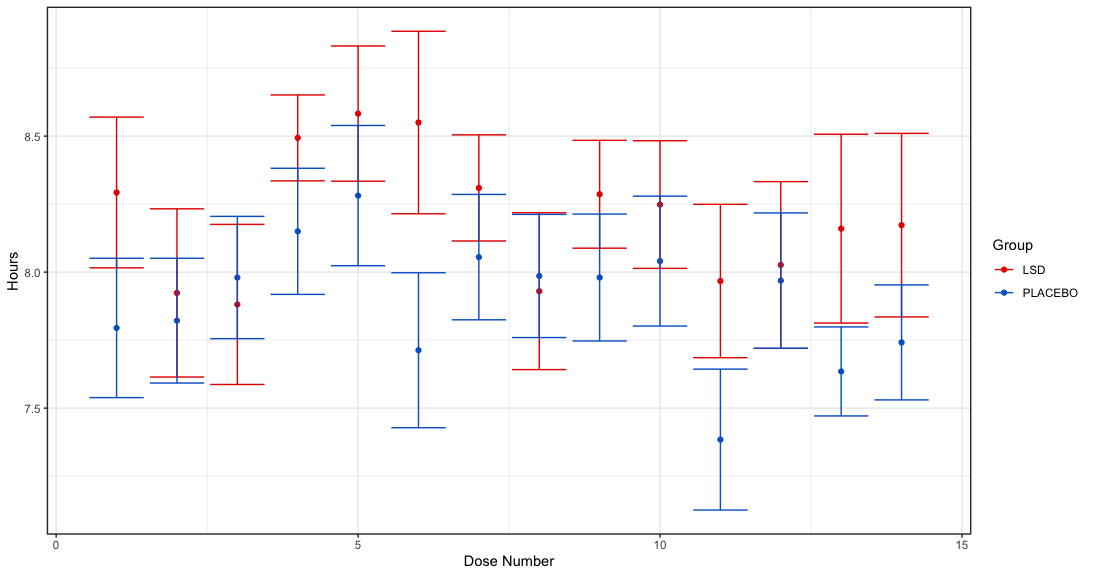
**

*Supplementary Figure 3: Grand average time spent in total sleep for each dose and group in the trial for the dose + 1 night. Error bars represent the standard error of the mean calculated across participants.*


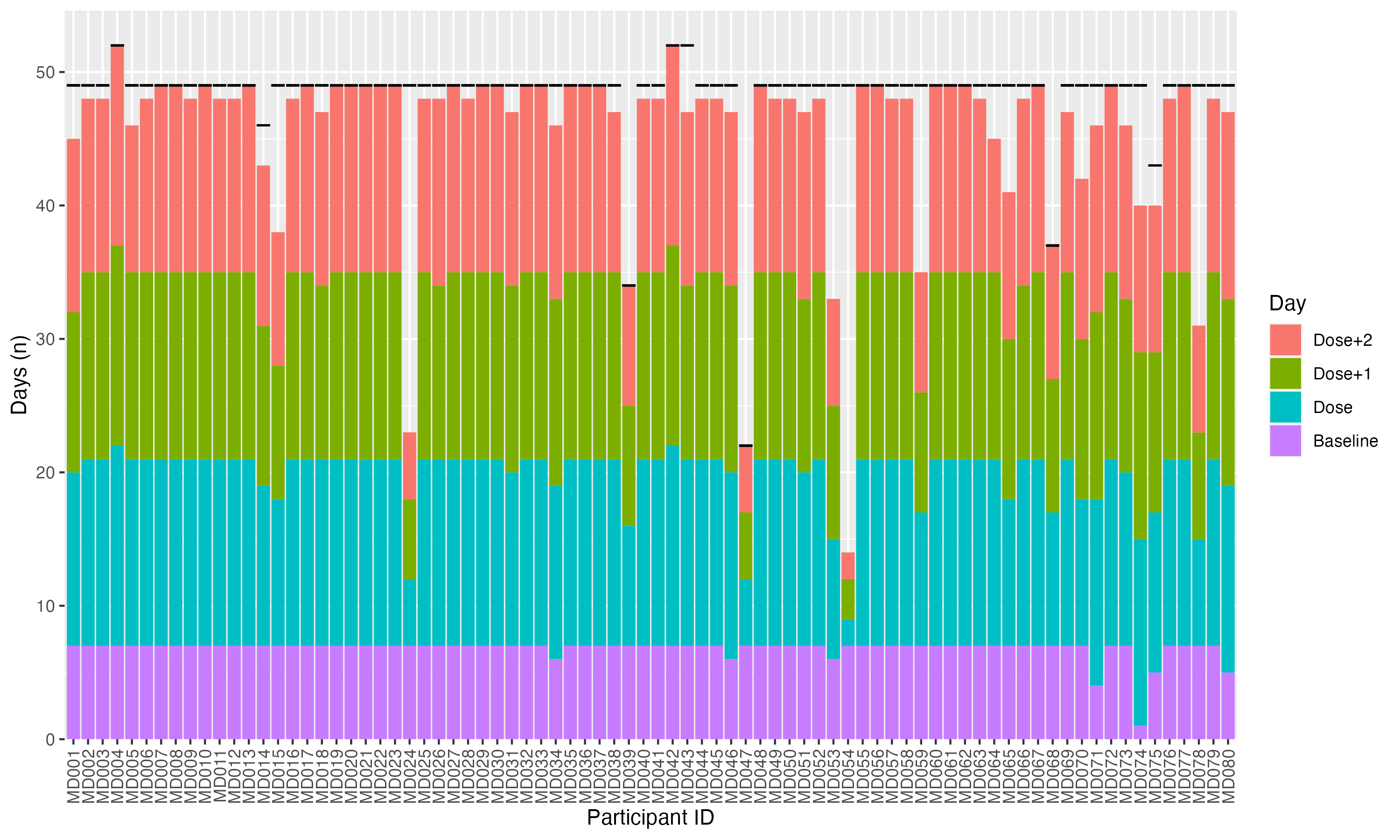


*Supplementary Figure 4: The number of days with quantifiable step data for each participant (MD001-MD080) characterised by baseline, dose day , dose+1 day, dose+2 day.*

**Participant Quotes**

*Decreased energy*

1. I do find on those dose days, that as I might have mentioned, I have lower energy, and my body feels a bit- like even, even when I mentally feel stimulated, my body felt a bit weak kind of throughout the day. So on those dose days, I was always a lot less motivated to do like a workout [Participant A, LSD]

2. Right. I think as far as energy level goes, like, definitely post-dosage I could definitely feel a low and I just felt more tired as if the dosing consumed a higher level of energy than normal. And so yeah, yeah just a little fatigue.[…] But particularly the next day afterwards. Yeah. But I think by the second or third day, I was normalising […] I definitely noticed extra fatigue for sure….For me, I would notice it the next morning, yeah. The same night it's a bit hard to differentiate, but that's just the evening fatigue. But the next morning is definitely, I can notice like, I slept like- I felt like I needed, like, to sleep in and stuff. [Participant B, LSD]

3. But never, it was probably- it was, I found it quite emotionally draining. [...] I probably had less energy on the days that I took it actually. [...]it was just probably just like, more of a lack of focus [Participant C, LSD]

*Increased energy*

1. It gave me like a good energy I think. It made me want to do things and like yeah, just like get into stuff and just go and do all these things that I wanted to do. I found it yeah, definitely... it gives you an interesting clarity.[…] And took out any of the buzz that was in the background and you can just deal with stuff as it came. [Participant D, LSD]

2. Except from that, I did feel like the, in terms of like energy levels. I mean, I mean, physical energy, I did feel when increase that. So I would maybe prefer, in the dose days do some maybe physical activities […] because it felt, that I had that extra energy. [Participant E, LSD]

3. Yeah, so... I felt very, like it was interesting, because I had like, huge rushes of energy Or I found myself- and maybe it's something to do with like the energy I felt- I found myself staying up later than I kind of wanted to. But equally, I didn't feel overly unrested the next day. Like, I know, I normally needed more sleep than I actually got often. But yeah, I didn't feel overly tired. [Participant F, LSD]
